# The Effect of Oral Probiotic *Bacillus amyloliquefaciens* on Intestinal Microbiota, Intestinal Structure, Serum Antioxidant Capacity and Inflammatory Responses of Heat Stressed Rats

**DOI:** 10.4014/jmb.2505.05008

**Published:** 2025-09-23

**Authors:** Xiaodong Zhang, Li Tang, Bluefin masell Freeman, Baikui Wang, Wenying Shen, Weifen Li

**Affiliations:** 1 College of Life and Environmental Sciences, Shaoxing University, Shaoxing 312000, P.R. China; 2 Key Laboratory of Molecular Animal Nutrition of the Ministry of Education, Institute of Feed Science, College of Animal Sciences, Zhejiang University, Hangzhou 310058, P.R. China

**Keywords:** *Bacillus amyloliquefaciens*, heat stress, intestinal microbiota, intestine morphology, inflammatory responses, serum antioxidant capacity

## Abstract

This study investigated the effect of *Bacillus amyloliquefaciens* (*BaSC06*) in alleviating heat stress in rats. The rats were randomly divided into four groups. Each rat in Group 1 and Group 3 received oral 1 ml/day of PBS, whereas those in Group 2 and Group 4 were administered 1 ml/day of *BaSC06* (10^8^ CFU/ml). After two weeks, the rats in Groups 3 and 4 were exposed to heat stress at 42°C for 30 min, while those in Groups 1 and 2 were maintained at 25°C. The result showed that oral *BaSC06* increased abundance of *Firmicutes* phylum and *Ruminococcaceae* Family along with reduced level of *Bacteroidetes* family. Compared to Group 1, the height of small intestine villi increased significantly (*P* < 0.05) in Group 2, and intestinal villus height and mucosal thickness decreased significantly in Group 3 than that in Group 4 (*P* < 0.05). The distribution of ZO-1 was disrupted in Group 3 which was ameliorated in Group 4. The number of bacteria in the liver, spleen and mesenteric lymph nodes was significantly higher in Group 3 than other groups (*P* < 0.05) meanwhile the LPS level in Group 4 decreased significantly compared to Group 3 (*P* < 0.05). The levels of IL-1, IL-10 , HSP70 and MDA increased significantly (*P* < 0.05), the activity of CAT decreased significantly (*P* < 0.05) in Group 3 compared to the other groups. The adverse effects of heat stress can be alleviated by gavage *BaSC06*, which improve the composition of intestinal microbiota. It is helpful to elucidate the mechanism of probiotics to counteract the negative effects of heat stress.

## Introduction

Temperature poses a significant challenge to both human and animal health, particularly in the animal husbandry industry, where heat stress adversely affects animal health and reduces productivity [1]. Heat stress damages intestinal histology and barrier function, resulting in enhanced intestinal permeability and the dissemination of bacterial endotoxin from the intestinal lumen into the bloodstream[2]. Furthermore, it may trigger a series of inflammatory responses due to an increase in humoral factors associated with inflammation in the blood [3]. Also, heat stress can result in the increasing expression of heat shock proteins (HSPs), which allow the body to adapt quickly to environmental changes [4]. Various stresses can disrupt the balance of intestinal microbiota which is vital to maintain intestinal mucosal barrier function, leading to reduce in organism immunity [5, 6]. Conversely, a stable intestinal microbiota helps tissues to deal with stress [7]. Therefore, regulating the intestinal microbiota may mitigate the damage induced by stressors like pressure and high temperature.

Adequate administration of probiotics is beneficial to promote intestinal health and keep intestinal homeostasis [8, 9]. Recently, probiotics have gained widespread use in animal husbandry to mitigate heat stress, improve intestinal permeability, and strengthen the intestinal barrier through balancing intestinal microbiota [10, 11]. Administration of probiotic combination increased the growth of broiler chickens which was related to an increase in relative abundance of *Bifidobacterium* spp., *Lactobacilli*, and Gram-positive cocci [12] and reduced harmful bacterial numbers in the intestines of pigs [13, 14]. The probiotic strains of *Lactobacillus agilis* JCM 1048 and *Lactobacillus salivarius* JCM 1230 could restore the microbial balance and maintain the natural stability of indigenous bacterial microbiota following heat stress-induced changes [15]. Song *et al*., [16] found that heat stress decreased the viable counts of *Lactobacillus* and *Bifidobacterium* while increasing the viable counts of *Coliforms* and *Clostridium* in small intestinal contents of broilers with shorter jejunal villus height, deeper crypt depth, increased jejunal paracellular permeability, and downregulated protein levels of occludin and zonula occludens-1(ZO-1). This effect was improved by supplementation of the probiotic mixture containing *Bacillus licheniformis*, *Bacillus subtilis*, and *Lactobacillus plantarum*. Moore *et al*., [11] revealed that oral administration of *B. subtillus* BSB3 strain can relieve the adverse effects related to heat stress in rats. Currently, there are few studies to clarify the mechanisms of probiotics alleviating heat stress.

As a probiotic, *Bacillus* can protect intestinal epithelial cells from damage and maintain intestinal homeostasis [17]. *Bacillus amyloliquefaciens* has been extensively utilized as a probiotic for decades, playing a significant role in human health, agriculture, and environmental protection, where it contributes to the production of beneficial substances and the inhibition of pathogens [18, 19]. Recent studies have focused on the antifungal proteins or antimicrobial factors from *B. amyloliquefaciens* [20, 21]. Previous studies in our lab found that *BaSC06* could increase antioxidant capacity, immune function and improve the intestinal microbiota composition in broilers and pigs [6, 22, 23]. This study aims to investigate the effects of the probiotic *BaSC06* on the intestinal microbiota composition, intestinal histology and humoral factors in rats exposed to heat stress.

## Materials and Methods

### *BaSC06* Bacterial Strain Preparation

The probiotic of *B. amyloliquefaciens* SC06 (*BaSC06*) was isolated from soil and stored in China Center for Type Culture Collection (No. M 2012280). The bacteria was cultured according to the method described by Cao X *et al*.,[24]. The purity and type of the bacteria were constantly checked using the spread plate method and diluted with sterile PBS to a concentration of 10^8^ CFU/ml and stored at 4°C for subsequent experiments.

### Rats Management

ICR rats were obtained from Experimental Animal Center of Zhejiang Province. This study was approved by and performed according to the guidelines of the Animal Care and Use Committee of Zhejiang University. Twenty-four male rats with average initial weight of 227.8 ± 3.3 g were randomly divided into four groups of equal size. Before the experiment, the rats were given ad libitum food and water and kept at room temperature for one week to adapt to the environment. The gavage experiments was performed after weighing. In group 1 and group 3, each rat was orally administrated with 1 mL of PBS per day, while in group 2 and group 4, each rat administrated with 1 ml of *BaSC06* (10^8^ CFU/ml) per day. After 2 weeks, the rats in group 3 and group 4 were exposed to heat stress treatment at 42°C for 30 min and 55% relative humidity, while group 1 and 2 were kept at temperature of 25°C and 55% relative humidity as control.

### Sample Collection

The rats were sacrificed by decapitation, and their serum was prepared by centrifugation at 5,000 r/min for 20 min before being stored at -20°C. Samples of the jejunum and ileum from each rat was collected and fixed with 10% formalin for histological analysis. Furthermore, samples of mesenteric lymph nodes, liver and spleen were aseptically taken for bacterial translocation analysis. Finally, faeces were collected for analysis of fecal microbial communities.

### Fecal DNA Extraction and Illumina Miseq Sequencing

The genomic DNA of fecal microbes was extracted by TIANamp Stool DNA Kit (Tiangen, China) and fecal microbial communities were investigated using 16S rDNA sequencing by targeting 16S rDNA V3/V4 hypervariable region, which was performed on an Illumina MiSeq platform (Illumina Inc., USA). Quality-filter of the paired-ends raw sequences and cluster of the filtered sequences into operational taxonomic unit (OTU) at 97% similarity were performed by QIIME software (v 1.9.1) [25]. Bacterial OTU representative sequences were assigned to a taxonomic lineage by RDP classifier based on the SILVA 132 release database. Difference analysis of microbial communities based on OUT levels between groups were calculated using “DESeq2” package and were visualized by “UpSetR” and “ggplot2” packages. The significant differences in bacterial taxonomies were analyzed and visualized by linear discriminant analysis effect size (LEfSe) analysis (https://huttenhower.sph.harvard.edu/galaxy/) and statistical analysis of taxonomic and functional profiles (STAMP) software with a two-sided Welch’s *t*-test [26].

### Bacterial Translocation

Samples of mesenteric lymph nodes, liver and spleen were collected and cut into small pieces. These pieces were then placed in a solution of 1 ml of PBS per gram of tissue to fully extract any bacteria present in the tissue. The solution was spread onto LB solid plates and incubated under both aerobic and anaerobic conditions at 37°C for 24 h. The total number of viable aerobic and anaerobic bacteria was then presented as CFU per gram of tissue (CFU/g).

### Intestinal Histology

The paraffin sections of 5 μm thick were prepared and subjected to Hematoxylin and Eosin staining. The height of the small intestine villi and crypt, the thickness of the mucosa, were observed using light microscope (Nikon Eclipse 80i, Japan) and measured using a micrometer. Then the height of the villi/the depth of the crypt (V/C) ratio was calculated.

Intestinal tight junction protein ZO-1 expression was analyzed using immunohistochemical streptavidin biotin-peroxidase complex method. The fixed paraffin sections were dewaxed and dehydrated then treated with hydrogen peroxide (H_2_O_2_) solution to block endogenous peroxidase activity. The sections were then incubated with rabbit anti-ZO-l polyclonal antibody (1:200) overnight at 4°C and subsequently incubated with HRP-labeled goat anti-rabbit IgG (1:200) at 37°C for 20 min. DAB (diaminobenzidine) staining was used to develop the sections, which detect the active site of peroxidase in cells. Finally, the sections were observed using microscope (Nikon Eclipse 80i, Japan).

### Cytokine Assay

The levels of interleukin(IL)-1, IL-10, endotoxin (LPS), and heat stress protein 70 (HSP70) in the serum were measured using enzyme-linked immunosorbent assay (ELISA) kits purchased from Shanghai Enzyme Biotechnology (China), following the manufacturer's instructions.

### Antioxidant Factors Assay

The levels of superoxide dismutase (SOD), glutathione (GSH), catalase (CAT), malonaldehyde (MDA) activities were analyzed using assay kits from Nanjing Jiancheng Bioengineering Institute (China), following the manufacturer's instructions.

### Data Analysis and Statistics

The data were presented as means ± standard deviation, and univariate analysis was conducted using SPSS 24.0 statistical software. Tukey’s multiple comparisons were utilized to compare the mean values between the groups. *P* < 0.05 was considered as statistically significant.

## Results

### Effect of *BaSC06* on Intestinal Microbiota Composition

**Fig. 1:**
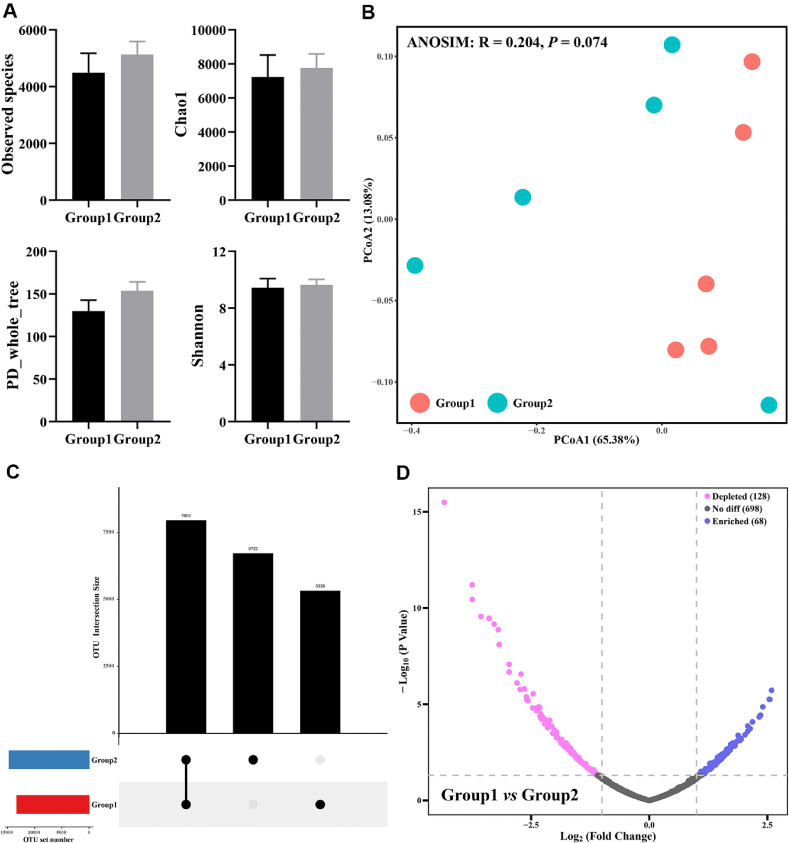
Difference analysis of intestinal bacterial community in rats. (**A**) Alpha diversity analysis. (**B**) Principal coordinates analysis (PCoA) based on Bray-Curtis distance. (**C**) UpSetR plot of the absolute OTU abundances among two groups. (**D**) Volcano plot of the fold changes of taxonomic abundances between two groups. Rats of Group 1 and Group 2 were pretreated with PBS or *Bacillus amyloliquefaciens* SC06 by oral gavage for 2 weeks.

The change of intestinal bacterial diversity with BASC06 supplementation was investigated using the 16S rRNA sequencing. The alpha diversity of the intestinal microbiota community was measured by Shannon, PD-whole-tree, Chao1 and Observed species indexes. It showed that no significant difference was observed between group 1 and group 2 (*P* > 0.05, Fig. 1A). Additionally, PCoA analysis revealed that the microbial community in Group 2 was no significant difference (*P* = 0.074) from the group 1 (Fig. 1B). Nevertheless, the UpSetR plot, which visualizes the intersections of sets based on absolute OUT levels, indicates that two groups share 7955 OUTs, with group1 and group2 containing unique OUTs of 6722 and 5328 respectively (Fig. 1C). A comparative analysis of bacterial community structures revealed that probiotic pretreatment in group 2 led to a significant increase in the abundance of 68 taxa and a significant decrease in 128 taxa, as compared to group 1 (Fig. 1D). These results indicated that the oral gavage of BaSc06 may not affect the diversity of bacterial community but alter microbial composition.

**Fig. 2:**
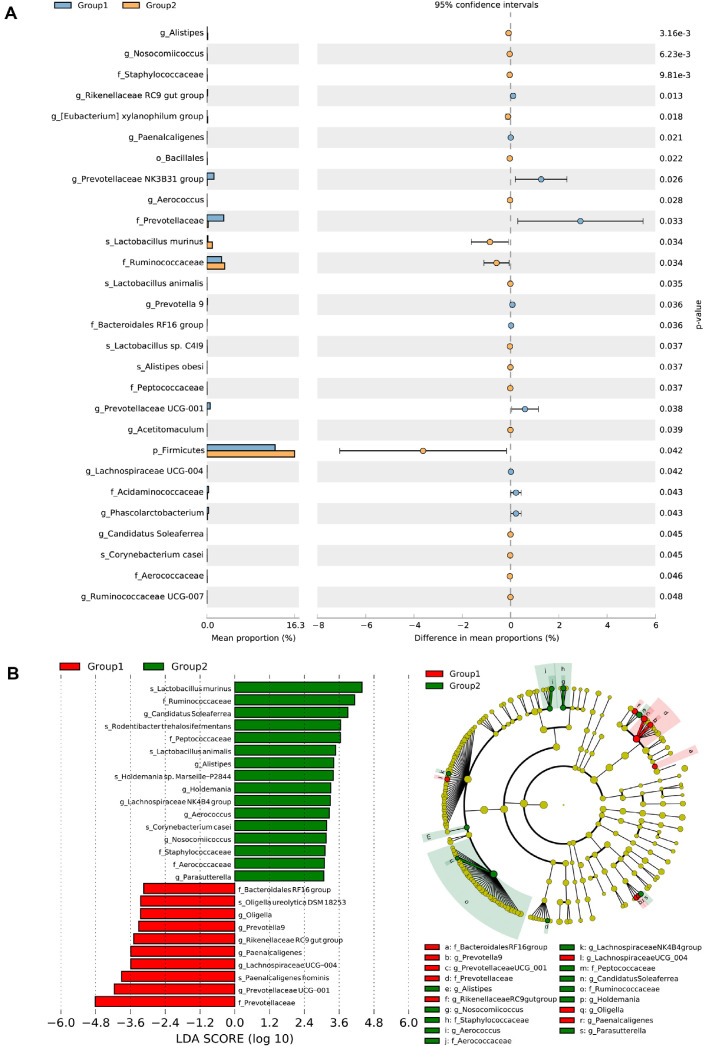
Comparison of the intestinal microbes. (**A**) Statistical analysis of taxonomic and functional profiles (STAMP), All results are expressed as mean±SD in each group. (**B**) Linear discriminant analysis (LDA) effect size (LEfSe) analysis (LDA > 2.4, *P* < 0.05). The prefix letters represented the taxonomy of the bacteria: p, phylum, c, class; o, order; f, family; g, genus. Rats of Group 1 and Group 2 were pretreated with PBS or *Bacillus amyloliquefaciens* SC06 by oral gavage for 2 weeks.

The distinct taxonomy taxa were further analyzed by statical analysis of metagenomic proﬁles (STAMP) multiple-test correction (Fig. 2A). *SC06* pretreatment increased significantly (*P* < 0.05) the relative abundance of g_*Alistipes*, g_*Nosocomiicoccus*, f_*Staphylococcaceae*, g_*[Eubacterium] xylanophilum* group, o_*Bacillales*, g_*Aerococcus*, s_*Lactobacillus murinus*, f_*Ruminococcaceae*, s_*Lactobacillus animalis*, s_*Lactobacillus* sp. C4I9, s_*Alistipes obesi*, f_*Peptococcaceae*, g_*Acetitomaculum*, p_*Firmicutes*, g_*Candidatus Soleaferrea*, s_*Corynebacterium casei*, f_*Aerococcaceae* and g_*Ruminococcaceae* UCG-007 compared with the group 1. Meanwhile the relative abundance of g_*Rikenellaceae* RC9 group, g_*Paenalcaligenes*, g_*Prevotellaceae* NK3B31 group, f_*Prevotellaceae*, g_*Prevotella* 9, f_*Bacteroidales* RF16 group, g_*Prevotellaceae* UCG-001, g_*Lachnospiraceae* UCG-004, f_*Acidaminococcaceae* and g_*Phascolarctobacterium* were reduced notably (*P* < 0.05).

Farthermore, LEfSe analysis results showed that 26 signiﬁcantly different taxa in the two groups were identified with LDA (score > 2.4, *P* < 0.05), mainly belonging to the phyla of *Firmicutes*, *Bacteroidetes*, *Proteobacteria*, and *Actinobacteria* (Fig. 2B). For group 2, s_*Lactobacillus murinus*, f_*Ruminococcaceae*, g_*Candidatus Soleaferrea*, s_*Rodentibacter trehalosifermentans*, f_*Peptococcaceae*, s_*Lactobacillus animalis*, g_*Alistipes*, s_*Holdemania* sp. *Marseille*-P2844, g_*Holdemania*, g_*Lachnospiraceae* NK4B4 group, g_*Aerococcus*, s_*Corynebacterium casei*, g_*Nosocomiicoccus*, f_*Staphylococcaceae*, f_*Aerococcaceae*, g_*Parasutterella* were predominant species. Group 1 exhibited enrichment of f_*Bacteroidales* RF16 group, s_*Oligella ureolytica* DSM 18253, g_*Oligella*, g_*Prevotella* 9, g_*Rikenellaceae* RC9 group, g_*Paenalcaligenes*, g_*Lachnospiraceae* UCG-004, s_*Paenalcaligenes hominis*, g_*Prevotellaceae* UCG-001 and f_*Prevotellaceae*. The results indicated that SC06 dramatically altered the gut microbiota, resulting in an elevated abundance of the *Firmicutes* phylum and *Ruminococcaceae* family, while simultaneously reducing the abundance of the *Bacteroidetes* family. In addition, we cultured the intestinal pathogenic bacteria using selective medium to analyze the effect of *B. amyloliquefaciens* SC06 on the harmful bacteria after heat stress. The result indicated the number of *Escherichia coli* significantly decreased?*p* < 0.05?, while the number of pathogenic *Vibrio* and *Pseudomonas* reduced in group 4, but there was no significant difference compared with that of group 3 (Table S1).

### Effect of *BaSC06* on the Histological Structure of Ileum

**Fig. 3:**
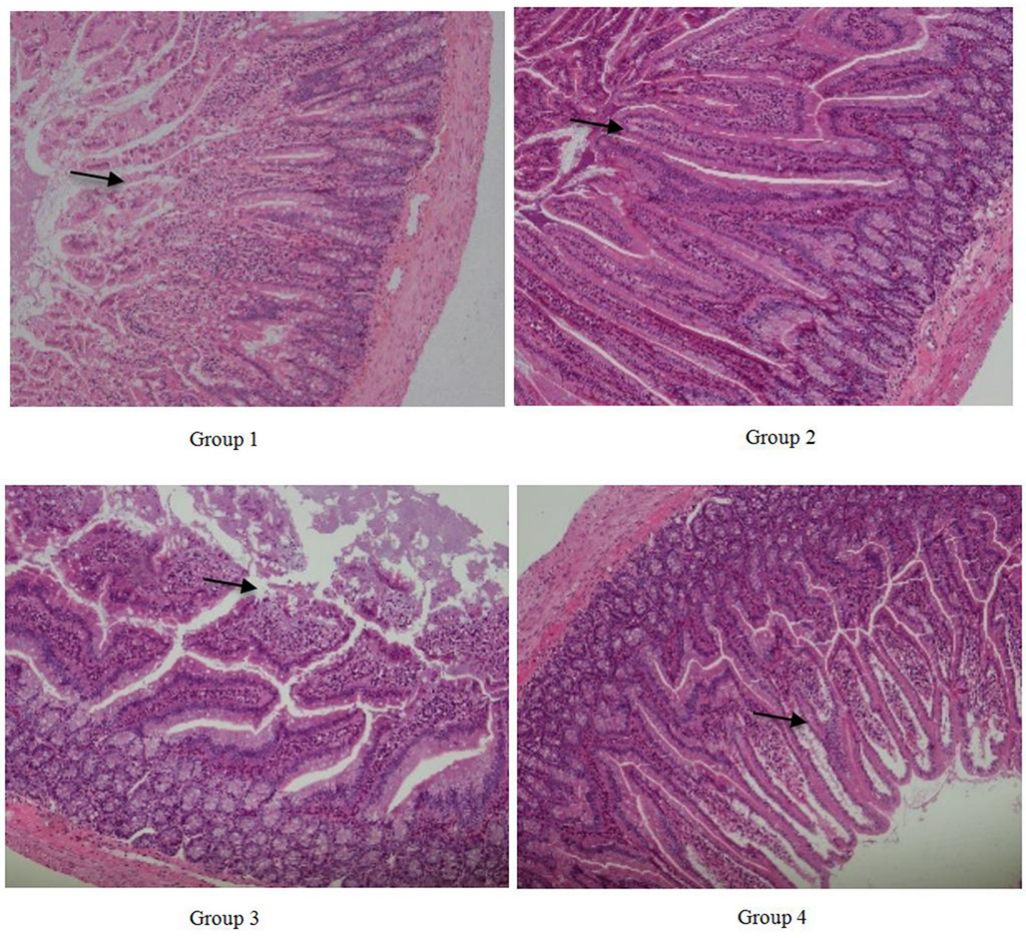
Effects of *Bacillus amyloliquefaciens* SC06 and heat stress on the histological structure of ileum. The black arrows indicated villi of ileum. Rats were pretreated with PBS (Group 1 and Group 3) or *Bacillus amyloliquefaciens* SC06 (Group 2 and Group 4) by oral gavage before exposure to 42°C or to 25°C.

The rats in groups 1 and 2, kept at a temperature of 25°C, exhibited normal ileum intestinal morphology. However, group 3 suffered severe damage to the villi and overall structure of the ileum due to heat stress. This damage was mitigated by the administration of *BaSC06* in group 4, as shown in Fig. 3.

The villus height in group 2 was significantly higher than that in group 1 (*P* < 0.05). The lowest villus height was found in group 3 and this difference was also significant (*P* < 0.05). When compared with group 2, there was no significant difference in villus height, crypt depth, V/C ratio and mucosal layer thickness in group 4. However, a significant increase in crypt depth and a decrease in V/C ratio was observed in group 3 when compared with group 4 (*P* < 0.05) (Table 1).

**Fig. 4:**
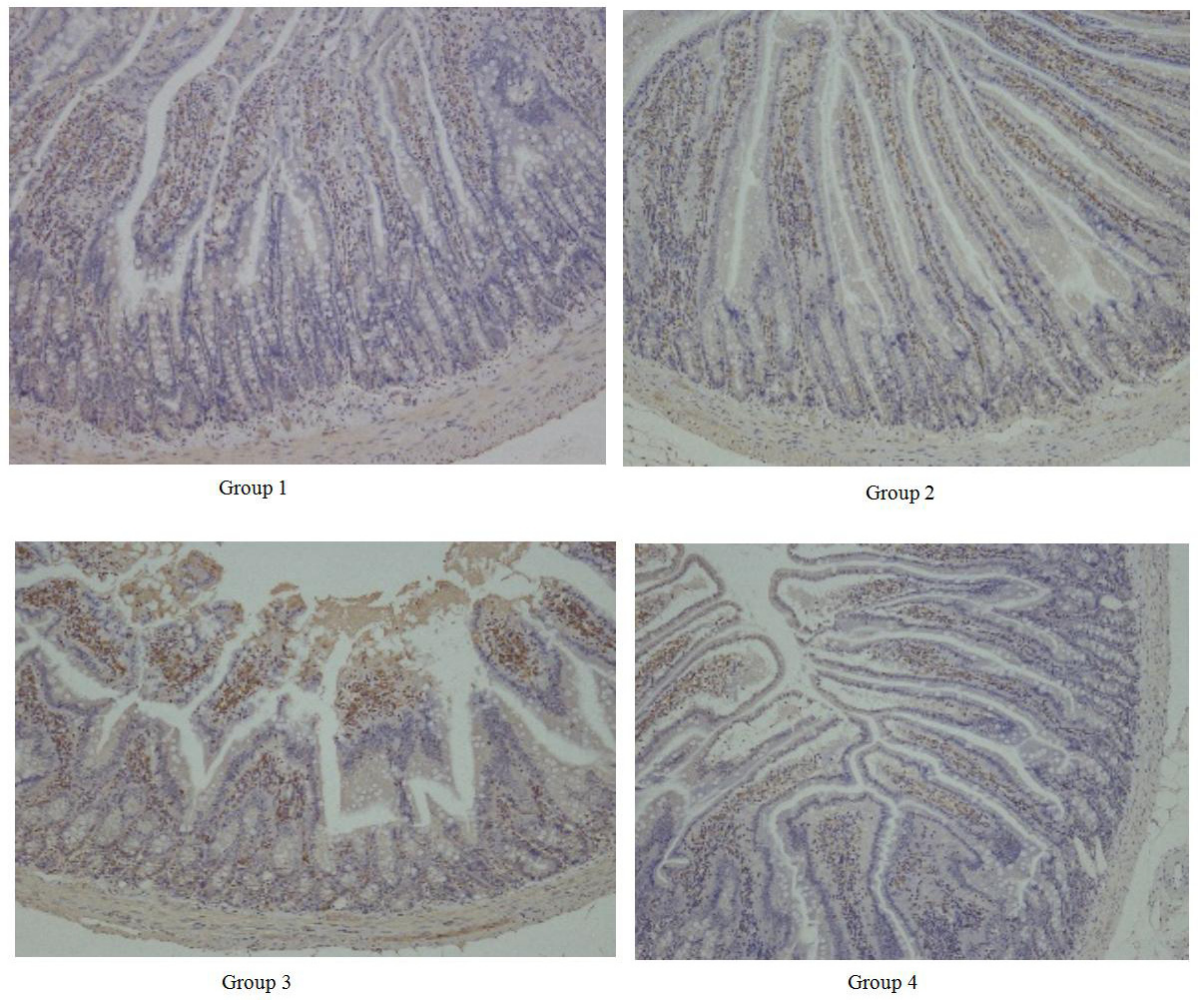
Effect of *Bacillus amyloliquefaciens* SC06 and heat stress on ZO-1 expression in ileum. ZO-1 expression was showed in brown dot. Rats were pretreated with PBS (Group 1 and Group 3) or *Bacillus amyloliquefaciens* SC06 (Group 2 and Group 4) by oral gavage before exposure to 42°C or to 25°C.

There was no significant alteration in the expression of tight junction protein ZO-1 in the ileum between group 1 and group 2 (*P* > 0.05). However, heat stress decreased significantly the expression of ZO-1 in group 3 and the distribution of ZO-1 among intestinal villus cells was not significantly disrupted in group 4 compared to group 3 (Fig. 4). These results indicated *BaSC06* treatment improved the intestine structure at room temperture, meanwhile reduced the damages caused by heat stress.

### Effect of *BaSC06* on Bacteria Translocation

**Fig. 5:**
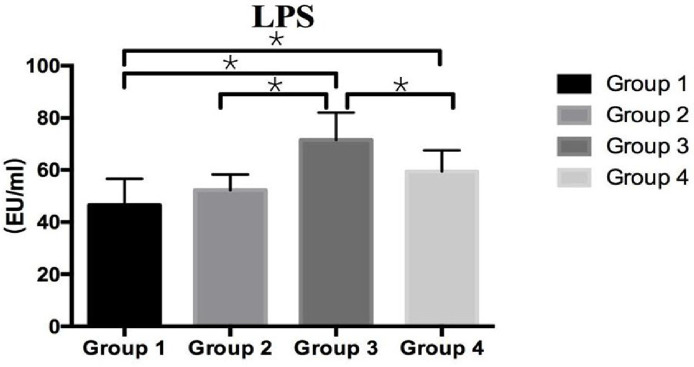
Effect of *Bacillus amyloliquefaciens* SC06 and heat stress on LPS level in serum. *P < 0.05 indicates significant difference. Rats were pretreated with PBS (Group 1 and Group 3) or *Bacillus amyloliquefaciens* SC06 (Group 2 and Group 4) by oral gavage before exposure to 42°C or to 25°C.

The numbers of bacteria present in the liver, spleen and mesenteric lymph nodes were no significant difference among group1, 2, and 4 (*P* > 0.05). However, there was highest level in group 3, which was higher than that in other three groups (*P* < 0.05) (Table 2). What’s more, LPS is widely considered as an important component of bacteria, and it was found that the LPS level was no siginificant variation between group 1 and 2, and a significant increase in group 3 compare to the other three groups (*P* < 0.05) (Fig. 5). The findings showed the administraton of *BaSC06* was no effect on the bacteria translocation at the room temperature, but could decrease the heat-stressed ralated translocation of gut bacteria and LPS.

### Effect of *BaSC06* on Humoral Factors and Antioxidant Factors

**Fig. 6:**
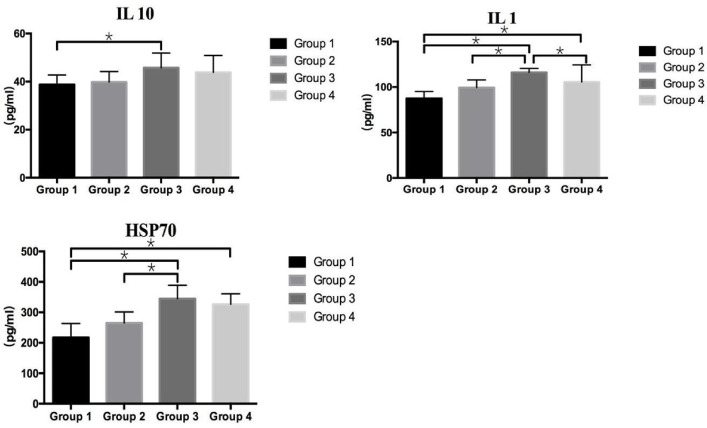
Effects of *Bacillus amyloliquefaciens* SC06 and heat stress on humoral factors in rats. *means significantly different at *P* < 0.05. Rats were pretreated with PBS (Group 1 and Group 3) or *Bacillus amyloliquefaciens* SC06 (Group 2 and Group 4) by oral gavage before exposure to 42°C or to 25°C.

The levels of IL-1, IL-10 and HSP70 were no siginificant variation between group 1 and group 2, whereas significantly (*P* < 0.05) increased in heat stress groups. However, the IL-1 level in group 4 decreased significantly compared to group 3 (*P* < 0.05) (Fig. 6). The activity of antioxidant enzymes (CAT, SOD, GSH) did not show a significant difference between group 1 and group 2. However, there was a noticeable increased MDA content (*P* < 0.05) and a reduced CAT activity (*P* < 0.05) in group 3 compared to other three groups (Table 3). The results revealed the gavage *BaSC06* could reduce inflammatory responses and enhance antioxidant capacity in heat stressed rats but no effect in normal ones.

## Discussion

It is widely recognized that intestinal microbiota, particularly probiotics, play a crucial role in promoting host immune system and animal health due to their diversity and functions [27]. Probiotics are frequently utilized as additives in animal feed, providing multiple advantages to the host. They not only help regulate intestinal homeostasis, but also enhance intestinal health. Probiotics hinder the overgrowth of pathogenic bacteria and the infiltration of external pathogens through diverse mechanisms, including as interfering with pathogens, improving barrier function, immunomodulation and neurotransmitter production [24, 28]. Different probiotic colonizations have a specific impact on intestinal microbiota composition in different species, indicating that these influences are species-specific. In this study, *BaSC06* did not affect the diversity of the intestinal microbiota but improved microbiota composition by significantly increasing the abundance of *Firmicutes* phylum and *Ruminococcaceae* family along with reduced the abundance of *Bacteroidetes* family. The findings consistent with earlier research conducted in pigs [29]. *Firmicutes* was mainly composed of *Bacillus*, *Lactobacillus* and other beneficial bacteria. These bacteria play a crucial role in sustaining intestinal health [30]. The latest research revealed the ratio of *Firmicutes*/*Bacteroidetes* is related to the transport and utilization ability of host nutrients [31]. SC06 notably elevated the ratio of Firmicutes to Bacteroidetes, suggesting that *SC06* was beneficial for the dietary utilization. *Ruminococcaceae*, which is one of the most abundant symbiotic bacteria in the healthy animal intestinal microbiota, has a variety of immune potentials and anti-inflammatory activity [32]. Multiple studies have proved the increased abundance of Ruminococcus could promote the growth of beneficial bacteria [33, 34], accelerate the production of butyrate [35, 36] and reduce the burden and translocation of lipopolysaccharide in the intestine [37, 38]. Therefore, probiotic *BaSC06* could strengthen the dominant position of the *Firmicutes*, and reduce gram-negative pathogen infection, to promote the intestinal microbial balance and microbial barrier function, thereby, alleviating damages caused by heat stress in rats.

*B. subtilis* is able to protect intestinal epithelial cells from damage and maintain intestinal homeostasis [17]. Heat stress can cause severe damage to intestinal morphology, manifested as shorter villi, deeper crypts, reduced V/C ratio, and thinner mucosa [28]. Crypt depth is one of the important indicators reflecting the renewal ability of intestinal mucosal cells. The shallower the crypt depth, the faster the renewal of mucosal epithelial cells. It is important to maintain an appropriate crypt depth in the intestine, which may help to remain the stability of intestinal morphology [29]. Nevertheless, heat stress can inhibit the regeneration of mucosal epithelial cells, leading to deeper crypts. Similar results have been observed in studies on the intestinal structure and function of heat-stressed pigs [39]. Alterations in gut microbiota can similarly induce changes in intestinal structure [27]. It has been reported that probiotic *B. licheniformis* can improve egg production, intestinal morphology, and mucosal immune function damaged by heat stress in laying hens [10]. Moore T *et al*. [11] found that treating with *B. subtilis* strain BSB3 before heat stress could prevent its damaging effects on the intestine, the height of villi and total mucosal thickness index of rats exposed to high temperature treated with *B. subtilis* were similar to those of control and non-stressed rats. This experiment also found that *BaSC06* reduced the damage to intestinal tissue morphology caused by heat stress and alleviated its adverse effects to some extent.

Intestinal epithelial barrier comprises a monolayer of enterocytes and tight junctions, controlling molecular transport via transcellular and paracellular routes [40]. Dysfunction of the intestinal tight junction barrier, along with increased permeability, permits the passage of luminal antigens, endotoxins, and bacteria into the bloodstream [41]. One major functional regulatory protein of tight junctions is the ZO-1 [42]. Previous studies have shown that heat stress can reduce the expression of ZO-1 in the jejunum of chickens [16] and dairy cows [43] to aggravate damage to the intestinal mucosal barrier. This study reveals that heat stress not only reduces the expression of ZO-1 but also changes its structural distribution. However, pretreatment with *BaSC06* can markedly elevate the expression of ZO-1 in heat stress rats, suggesting a beneficial impact on intestinal tight junctions.

Heat stress not only leads to abnormal intestinal morphology and damage to the intestinal barrier, but also trigger the translocation of intestinal bacteria to the liver, spleen, and other tissues. Furthermore, it increases epithelial permeability, thereby intensifying the shift of bacteria and LPS into the bloodstream [44, 45]. In this experiment, the total amount of bacteria in the liver, spleen and mesenteric lymph nodes and serum LPS level in group 3 were significantly higher than those in the other three groups (*P* < 0.05). It has been reported pretreatment with *B. subtilis* strain BSB before heat stress can effectively alleviate bacterial translocation by maintaining normal intestinal structure in rats [11]. *BaSC06* could decrease bacterial translocation in weaned mice correlated with an increase in macrophage numbers [46]. This study found that pretreatment with *BaSC06* effectively alleviated bacterial translocation and LPS level in serum of rats exposed to heat stress. The reduced translocation of gut bacteria and LPS was closely related to improved intestinal microbiota composition, microbial barrier function, intestinal tight junction and the integrity of the intestinal structure with oral administration of Ba SC06.

In a high-temperature environment, IL-1 acts on the hypothalamus, which is regarded as the body's temperature regulation center. Elevated body temperature results in fever, triggering the upregulation of HSP70 expression to counteract high temperature damage and sustain normal cellular metabolic activity [47]. Moreover, up-regulation of HSP70 expression has a significant anti-inflammatory effect by reducing the production of inflammation-related cytokines and increasing the expression of the anti-inflammatory factor IL-10 [48]. Elevated IL-10 levels can markedly diminish the inflammatory cascade [49]. Our study further revealed significant elevation levels of HSP70 and IL-1 in the heat stress groups. Administration of *B. amyloliquefaciens* was observed to lower IL-1 levels, while unaffect IL-10 levels. Zhang *et al*. [50] discovered that *B. subtilis* HDaBS1 can alleviate heat stress response in rats by reducing IL-1 expression while increasing IL-10 expression. These inconsistent results may be attributed to differences between species and studies concerning the duration and severity of heat exposure.

The levels of oxidation and reduction in the body normally maintain a dynamic balance which can be broken by harmful endogenous and exogenous stimuli, such as high temperature [51]. The excessive generation of free radicals and reactive oxygen species (ROS) can decrease the body antioxidant capacity, elicit antioxidant defense mechanisms [52, 53]. Free radicals can cause peroxidative damage to cell membrane lipids and produce MDA that attacks polyunsaturated fatty acids in biological cell membranes, further triggering lipid peroxidation to cause more severe oxidative stress damage [54]. Within the antioxidant system, SOD, GSH, and CAT are most important antioxidant enzymes that resist excessive ROS [55, 56]. Probiotics can alleviate oxidative stress by increasing antioxidant capacity. Dietary supplementation of *Streptococcus faecalis* can significantly increase the hepatic activities of SOD, CAT, and GPx, while liver MDA content and plasma NO levels are opposite [57]. In this study, *BaSC06* can increase the antioxidant capacity and alleviate heat-induced oxidative stress, as evidenced by increased CAT activity and decreased MDA levels in rat serum.

In conclusion, oral administration of *B. amyloliquefaciens* SC06 can improve the composition of intestinal microbiota to enhance microbial barrier function and maintain the integrity of the intestinal structure, thereby reducing bacterial translocation and LPS level to decrease inflammatory responses and increase the serum antioxidant capacity. *BaSC06* can alleviate adverse reactions caused by heat stress and prevent heat stress-related complications in rats. Therefore, administering probiotic *BaSC06* may serve as an efficacious approach to mitigating the detrimental effects of heat stress. However, further research on *BaSC06* should be performed to determine its impacts on intestinal immune function and injured intestinal repair in heat stressed rats. However, more convincing results should be further investigated to confirm the transient or stable colonization status of *BaSC06* in intestine, such as by integrating mCherry or GFP fluorescent proteins into *BaSC06* bacterial genome, which would help elucidate the beneficial effects of *BaSC06*.

## Supplemental Materials

Supplementary data for this paper are available on-line only at http://jmb.or.kr.



## Figures and Tables

**Table 1:** Effects of *Bacillus amyloliquefaciens* SC06 and heat stress on intestinal villus height, crypt depth and mucosal layer thickness.

Groups	Villus height (μm)	Crypt depth (μm)	Villus height / crypt depth (V/C)	Mucosal layer thickness /μm
Group 1	416.22 ± 15.15^b^	88.68 ± 15.50^b^	4.59 ± 0.63^a^	383.96 ± 16.29^a^
Group 2	479.89 ± 27.87^a^	111.28 ± 17.53^b^	4.16 ± 0.31^a^	361.13 ± 19.54^a^
Group 3	354.97 ± 8.43^c^	168.51 ± 54.22^a^	2.31 ± 0.71^b^	306.23 ± 37.6^b^
Group 4	472.64 ± 42.31^a^	99.31 ± 13.67^b^	4.64 ± 0.47^a^	373.40 ± 13.85^a^

Different superscript letters of same column are significantly different at *P* < 0.05. Rats were pretreated with PBS (Group 1 and Group 3) or *Bacillus amyloliquefaciens* SC06 (Group 2 and Group 4) by oral gavage before exposure to 42°C or to 25°C.

**Table 2:** Protective effect of *Bacillus amyloliquefaciens* SC06 against bacterial translocation.

Groups	Liver	Spleen	Mesenteric lymph nodes
Group 1	121.5 ± 16.1^b^	55.0 ± 15.0^b^	60 ± 18.2^b^
Group 2	46 ± 12.3^b^	170 ± 20.0^b^	20 ± 5.6^b^
Group 3	555 ± 45.0^a^	1680 ± 90.0^a^	333 ± 25.0^a^
Group 4	87 ± 20.8^b^	315 ± 60.2^b^	40 ± 5.0^b^

Different superscript letters in the same column indicate significant differences (*P* < 0.05). Rats were pretreated with PBS (Group 1 and Group 3) or *Bacillus amyloliquefaciens* SC06 (Group 2 and Group 4) by oral gavage before exposure to 42°C or to 25°C. Homogenates of liver, spleen, and mesenteric lymph nodes were analysed for aerobic and anaerobic bacteria. Total bacterial count (aerobic and anaerobic) found in homogenates of each rat is presented as CFU per gram tissue (CFU/g).

**Table 3:** Effects of *Bacillus amyloliquefaciens* SC06 and heat stress on antioxidant factors in rats.

Group	CAT (U/ml)	SOD (U/ml)	GSH (U/ml)	MDA (nmol/ml)
Group 1	0.136 ± 0.023^ab^	5.721 ± 0.615^a^	686.275 ± 8.985^a^	16.138 ± 0.990^b^
Group 2	0.188 ± 0.043^a^	4.930 ± 0.241^a^	668.628 ± 45.691^a^	18.307 ± 0.730^b^
Group 3	0.090 ± 0.023^b^	7.511 ± 1.844^a^	656.863 ± 67.924^a^	22.472 ± 1.311^a^
Group 4	0.128 ± 0.047^ab^	5.956 ± 0.756^a^	611.765 ± 45.943^a^	11.955 ± 0.284^c^

Different superscript letters in the same column indicate significant differences (*P* < 0.05). Rats were pretreated with PBS (Group 1 and Group 3) or *Bacillus amyloliquefaciens* SC06 (Group 2 and Group 4) by oral gavage before exposure to 42°C or to 25°C.
