# Supplementary material for: The Effect of Oral Probiotic Bacillus amyloliquefaciens on Intestinal Microbiota, Intestinal Structure, Serum Antioxidant Capacity and Inflammatory Responses of Heat Stressed Rats
Source: J Microbiol Biotechnol. 2025 Sep 23;35:e2505008. doi: 10.4014/jmb.2505.05008 (PMC12535860; doi:10.4014/jmb.2505.05008)
Supplement: Supplementary file 1 [file jmb-35-e2505008-supple.pdf]

J. Microbiol. Biotechnol. 2025. 35: e2505008
https://doi.org/10.4014/jmb.2505.05008

Supplementary Table

Table S1. The effect of *Bacillus amyloliquefaciens* SC06 on the harmful bacteria in the intestine after heat stress. Different superscript letters of same column are significantly different at $P < 0.05$. Rats were pretreated with PBS (Group 3) or *Bacillus amyloliquefaciens* SC06 (Group 4) by oral gavage before exposure to 42°C. The bacterial count found in faeces is presented as CFU per gram tissue (CFU/g).

Selective medium	Pathogenic bacteria	Group 3 /x10^6	Group 4 /x10^6
TCBS	pathogenic *vibrio*	24.12 ± 9.11^a	16.01 ± 3.52^a
EMB	*Escherichia coli*	21.41 ± 7.39^a	1.20 ± 0.00^b
PSE	*Pseudomonas*	0.17 ± 0.09^a	0.04 ± 0.05^a
